# The Royal Army Medical Corps Section

**Published:** 1912-01-20

**Authors:** 


### THE ROYAL ARMY MEDICAL CORPS SECTION.

### Territorial Transport for Sick and Wounded.

Necessary as an efficient transport is for carrying out the military operations of an army, it is equally so for its medical service, for there fall to the latter the two very important duties of quickly transmitting the sick from the front to the rear, so as not to have an accumulation of non-effective men with the fighting force, and, secondly, of removing the wounded from the battlefield for conveyance to a clearing, stationary, or base hospital, as the case may be. In connection with the Territorial Force the plan decided on for dealing with the soldier from the time he falls wounded on the battlefield until he finds himself in a comfortable bed in the base or general hospital has been already outlined in The Hospital, and we need not go into the matter further beyond stating that it has been modelled on that of the Regular Army, with its three—(1) Collecting, (2) Evacuating, and (3) Distributing—zones. What we are desirous to draw attention to are the means by which the wounded soldier is moved from one to the other of these various stages through which he has to pass.

The regulation methods of medical transport are three in number, namely, (1) Stretchers, (2) Ambulance waggons, and (3) Hospital trains, and with all these our Regular Army is provided. To what extent have they been furnished to the Territorial Force? Taking a division as an illustration, and dealing first with the stretchers, the mobilisation tables show that the number of these, not including those belonging to the ambulance waggons, allotted to each division is 287. Of these 287 stretchers, 152 do duty with the infantry battalions of the division, twelve with the Mounted Brigade Field Ambulance, 108 with the Field Ambulance, twelve with the Yeomanry regiments, and three with the general hospitals. In peace time only 129 of the above 287 stretchers are issued for purposes of instruction, and they are distributed to the different branches in varying amounts, the infantry battalions having in charge seventy-six, the Mounted Brigade Field Ambulance six, the field ambulances thirty-six, the Yeomanry regiments eight, and the general hospitals three. They are all of the same pattern, namely, Mark I. and Mark II., the former, however, predominating. Mark II. is a later pattern and is gradually being issued instead of Mark I. It is very similar to it, except that the poles are fitted on the under side with steel V-shaped runners, instead of brackets with rollers, experience having shown that rollers are apt to break and split. Mark II. stretcher can be used with the ambulance waggons, which are adapted to receive them. Web slings are now being issued instead of the leather ones, as they are better for the men to carry with and they are more easily fixed on to the handles of the stretcher poles. Each stretcher has a wedge-shaped pillow of brown canvas, stuffed with horse-hair, but neither Mark I. nor II. has any recognised hood. If one was necessary, it would require to be improvised. The only Army stretcher with a recognised collapsible hood is the special Mark I., but we are not aware that any of these are in use in the Territorial Force. We understand a new Army stretcher, with a fixed canvas pillow, is contemplated, but it has not yet been issued. There are no cycle stretchers in use with the Territorial Force as was formerly the case with the Volunteers. The reason for this is that the military authorities do not recognise them and give no allowance for them, as they consider that the light ambulance waggon Mark I. of the Mounted Brigade Field Ambulance can do with mounted troops all that cycle stretchers are capable of, and as their utility with cavalry was the real justification for the use of cycle stretchers, it is a natural consequence that they should now be abandoned. If care is taken of the ordinary Army stretcher it is found to be a durable and efficient article. Its chief weakness is the splitting of the canvas after being in use for some time, and we suggest that it would be a good plan to supply a certain number of extra sheets of canvas of a proper size, along with copper nails and leather strappings, so as to carry out repairs expeditiously.

Passing next to the ambulance waggons of the Territorial Force, the mobilisation tables give a total of forty to each division, thirty-six being heavy waggons and four light ones. They are apportioned in the ratio of ten (four light and six heavy) to the Mounted Brigade Field Ambulance and thirty heavy ones to the three field ambulances. In peace time there are thirty held on charge for instructional purposes, along with the necessary harness and transport *personnel*, of which four (two light and two heavy) are with the Mounted Brigade Field Ambulance, and nine with each of the three field ambulances. The patterns usually issued are Mark I. (light) and Mark V.* (heavy). The Mark I. (light) is constructed to carry two patients on stretchers or eight sitting. The Mark V.* is a conversion of Mark V. and is built to carry four men on stretchers (two on the floor and two on the seats above) or twelve men sitting, six on each side. Another plan is to have two men on stretchers above the floor and four sitting at the rear end. Both Mark I. and Mark V.* are fitted with a quarter-lock or Jacob's lock fore-carriage, which reduces the strain on the body in travelling and admits of large front wheels being used, so as to minimise the pull on the horses. The problem in an ambulance waggon is to consider both horse and patient, and experience shows that the patient is often sacrificed in the matter of construction to the horse, with the result that the Army horse is rather a pampered creature and will not do the work of the civilian animal. There is in use in the Regular Army another waggon, Mark VI., with the same accommodation as Mark V.*, but it is a full-lock waggon with smaller wheels in front, thus allowing the pole to turn to any angle. It is the outcome of the public competition that took place in 1902 for the best

Army ambulance waggon, but none of these have been issued to the Territorial Force.

With this amount of equipment for the carriage of the wounded their needs should be amply met in the evacuating zone, and their removal from the battlefield carried out expeditiously and comfortably; but they cannot remain in the tent division of the field ambulance and they must be taken to the clearing or stationary hospitals, unless good railway accommodation exists close at hand, when they can go straight down by rail to the general hospitals. In the case of the Territorial Force, the duties of this evacuating zone are to be in the hands of the Voluntary Aid detachments, and it is necessary that they should have at their command proper carriage for the wounded. As yet nothing has been done by the Government in furnishing them with any such equipment, and there are in existence no hospital trains. The idea is that the members of these detachments are to be skilful in extemporising stretchers, in converting country carts into suitable conveyances, and in rendering available goods waggons on railways for the carriage of the wounded instead of hospital trains. No doubt a great deal can be done in this direction, but it is a point for consideration whether or not the provision of a certain amount of transport material for the wounded should not be placed at the command of the Voluntary Aid detachments, and some steps be taken in time of peace to arrange for the provision of well-appointed hospital trains, or, at any rate, to settle on the best plan of adapting the existing railway stock to the carriage of the sick and wounded, and have ready the materials required for doing so.

In another article we hope to return to this matter, as it is one of the greatest importance and should be thoroughly thought out, especially when it is remembered that in time of invasion the military authorities would have the first call on all horses and conveyances for use with the Army in the field. We understand that the British Red Cross Council purpose publishing a manual on the subject of extemporary transport. Such a publication should be of great service, and we hope its issue will be accelerated.

